# Mass spectrometry-based proteomics of cerebrospinal fluid in pediatric central nervous system malignancies: a systematic review with meta-analysis of individual patient data

**DOI:** 10.1186/s12987-024-00515-x

**Published:** 2024-02-13

**Authors:** Christian Mirian, Maria Thastrup, René Mathiasen, Kjeld Schmiegelow, Jesper Velgaard Olsen, Ole Østergaard

**Affiliations:** 1 Department of Paediatrics and Adolescent Medicine, Rigshospitalet, Copenhagen, Denmark; 2 Novo Nordisk Foundation Center for Protein Research, Faculty of Health and Medical Sciences, University of Copenhagen, Copenhagen, Denmark; 3 Institute of Clinical Medicine, University of Copenhagen, Copenhagen, Denmark

**Keywords:** Mass spectrometry, Proteomics, Cerebrospinal fluid, Pediatric, Leukemia, Central nervous system, Brain tumor, Meta-analysis

## Abstract

**Background:**

The cerebrospinal fluid (CSF) proteome could offer important insights into central nervous system (CNS) malignancies. To advance proteomic research in pediatric CNS cancer, the current study aims to (1) evaluate past mass spectrometry-based workflows and (2) synthesize previous CSF proteomic data, focusing on both qualitative summaries and quantitative re-analysis.

**Main:**

In our analysis of 11 studies investigating the CSF proteome in pediatric patients with acute lymphoblastic leukemia (ALL) or primary brain tumors, we observed significant methodological variability. This variability negatively affects comparative analysis of the included studies, as per GRADE criteria for quality of evidence. The qualitative summaries covered 161 patients and 134 non-tumor controls, while the application of validation cohort varied among the studies. The quantitative re-analysis comprised 15 B-ALL *vs* 6 “healthy” controls and 15 medulloblastoma patients *vs* 22 non-tumor controls. Certain CSF proteins were identified as potential indicators of specific malignancies or stages of neurotoxicity during chemotherapy, yet definitive conclusions were impeded by inconsistent data. There were no proteins with statistically significant differences when comparing cases versus controls that were corroborated across studies where quantitative reanalysis was feasible. From a gene ontology enrichment, we observed that age disparities between unmatched case and controls may mislead to protein correlations more indicative of age-related CNS developmental stages rather than neuro-oncological disease. Despite efforts to batch correct (HarmonizR) and impute missing values, merging of dataset proved unfeasible and thereby limited meaningful data integration across different studies.

**Conclusion:**

Infrequent publications on rare pediatric cancer entities, which often involve small sample sizes, are inherently prone to result in heterogeneous studies—particularly when conducted within a rapidly evolving field like proteomics. As a result, obtaining clear evidence, such as CSF proteome biomarkers for CNS dissemination or early-stage neurotoxicity, is currently impractical. Our general recommendations comprise the need for standardized methodologies, collaborative efforts, and improved data sharing in pediatric CNS malignancy research. We specifically emphasize the possible importance of considering natural age-related variations in CSF due to different CNS development stages when matching cases and controls in future studies.

**Supplementary Information:**

The online version contains supplementary material available at 10.1186/s12987-024-00515-x.

## Introduction

Cancer cell dissemination to and within the central nervous system (CNS) significantly increases the risk of treatment failure and remains a clinical challenge. The CNS is surrounded by cerebrospinal fluid (CSF) that circulates through the ventricles, cisterns, and subarachnoid space [1]. The CSF exerts cytotoxic properties, characterized by deprived or low levels of oxygen, nutrients and growth factors, that affect the survival of microorganisms and disseminated cancer cells [2]. Despite this, cancer dissemination within the CSF is a common occurrence in many pediatric patients.

Current analytical methods are inadequate for detecting cancer cells in the CSF, and establishing reliable biomarkers are warranted [3–7]. Multi-dimensional flow cytometric analysis of CSF indicate that 25–28% of children with acute lymphoblastic leukemia (ALL) have blast invasion at diagnosis, while the conventional cytomorphological examination shows only a 13–18% detection rate in contrast [8, 9]. For primary brain tumors like ependymoma and medulloblastoma, detection of circulating tumor cells relies on cytological examination of CSF, which often lacks specificity. Consequently, many childhood cancer patients undergo CNS-directed chemotherapy or radiotherapy, regardless of confirmed CNS dissemination. Such aggressive treatments carry a high risk of short- and long-term adverse events, that may significantly impact quality of life [10–15]. Reliable biomarkers to predict the risk of cancer dissemination and neurotoxicity could facilitate more tailored treatment intensity, yet such biomarkers are still to be established.

The CSF facilitate a functional compartment that is pivotal for the CNS microenvironment, and advancing CSF research through proteomics may offer important insight into the pathology of a range of CNS diseases. However, the technological advancement is rapidly developing while pediatric CNS cancer patients encompass a rare subgroup of patients. Consequently, the comparability between studies are often complicated by limited cohort sizes that are analyzed heterogeneously. Therefore, synthesizing evidence and experiences from previous studies is crucial to drive meaningful progress in this field of research.

Our primary objectives are twofold. Firstly, to compile and assess previous mass spectrometry-based workflows, here particularly focusing on the pre-analytical, analytical, and validation processes in existing literature. Secondly, to gather and examine evidence from prior research on CSF proteomics in pediatric CNS cancer patients, here summarizing findings qualitatively or re-analyzing available mass spectrometry data quantitatively when available.

## Methods

We adhered to the PRISMA-IPD guidelines (Preferred Reporting Items for Systematic Review and Meta-Analyses of individual participant data) [16].

### Population of interest

The targeted population encompassed childhood cancer patients with any malignancy involving the CNS. We sought to explore CSF proteome characteristics that could indicate disease presence or detection of neurotoxicity.

### Inclusion and exclusion criteria

Eligible studies included those published post-2000 that utilized a bottom-up mass spectrometry-based workflow. No restrictions were imposed on study design or language. All studies involving pediatric CNS cancers were considered relevant.

### Search strategies

We conducted a systematic search on November 9th, 2022, using PubMed, Google Scholar, and EMBASE. We included PubMed, Google Scholar, and EMBASE to ensure a broad and inclusive scope. While EMBASE may be preferred for its quality in biomedical literature, we also utilized PubMed and Google Scholar to capture a wider spectrum of relevant studies. Two authors, CM and RM, independently performed the search utilizing the Covidence platform. The following search string was used: (cerebrospinal fluid OR CSF) AND (proteom* OR mass spectrometry) AND (child* OR pediatric) AND (leukemia OR brain tumor OR malign* OR cancer).

### Study selection

Studies were selected based on adherence to our inclusion criteria. The primary outcomes of interest were the specifics of pre-analytical, analytical, and validation workflows in the selected studies, along with the qualitative and quantitative synthesis of CSF proteome alterations.

### Outcomes

The first outcome was to report the mass spectrometry-based workflows used in the included studies. The second outcome involved synthesizing and re-analyzing evidence from previous studies that employed this methodology in the specified patient group.

### Data extraction

Data extraction for the systematic review was conducted by author CM, focusing on candidate proteins reported in each study and with a "Reviewed" status in the UniProt database, which was verified manually from November 27th to 28th, 2022. This included information on key molecular functions, biological processes, and associations with diseases.

Subsequently, we extracted and summarized the sample preparation protocol (CSF collection method, CSF preparation prior to storage and storage conditions of the CSF samples), applied analytical workflow (CSF amount, discovery workflow and proteome depth) and validation (validation cohort, analytical technique and data availability) for each of the individual studies.

### Quality of evidence and bias assessment

The quality of evidence was assessed using the GRADE guidelines (Grades of Recommendation, Assessment, Development, and Evaluation) [17], with emphasis on inconsistency, imprecision, indirectness, and finally risk of bias [18–21].

### Quantitative synthesis: individual patient data meta-analysis

For quantitative synthesis, including a meta-analysis of individual patient data, we extracted raw mass spectrometry data files when available. Two authors, CM and OOE, collaboratively retrieved data from the relevant data repository. We analyzed the raw files extracted from each study and merged them if possible. We undertook a MaxQuant-search (v. 1.16.4.0 with *Match between runs*, no *Second peptides* and *label-free quantification mode*) using the SwissProt database FASTA file (downloaded 23rd January, 2021). We removed contaminants, reverse hits and hits identified only by site, were removed. Each identification was required as a valid value in 50% of the samples. All intensity values were subsequently log_2_-transformed and normalized using the LOESS method. Missing values were imputed based on values drawn from the normalized data. The datasets were kept separated based on cancer entity, which corresponded to ALL and brain tumors (other cancer types were not identified). We employed the Welch *t*-test to compare mean MS2-intensities for each protein intensities for each protein between malignant cases and control groups.

Further, we merged and batch-corrected the datasets using the HarmonizR approach [22], applying the ComBat algorithm to the log2-transformed MS2-intensities without imputation. We compared malignant cases and controls using a Welch *t*-test and rank orders.

Finally, we also conducted a gene ontology enrichment analysis to identify biological functions associated with the CSF-proteome, using uncorrected datasets. For this, we uploaded all quantified protein entries to the DAVID bioinformatics resources v. 2021 (The Database for Annotation, Visualization and Integrated Discovery) [23, 24]. The Benjamini–Hochberg method was utilized to adjust for the false discovery rate in *P*-values, here focusing on biological functions with corrected *P*-values below 0.01.

## Results

**Fig. 1:**
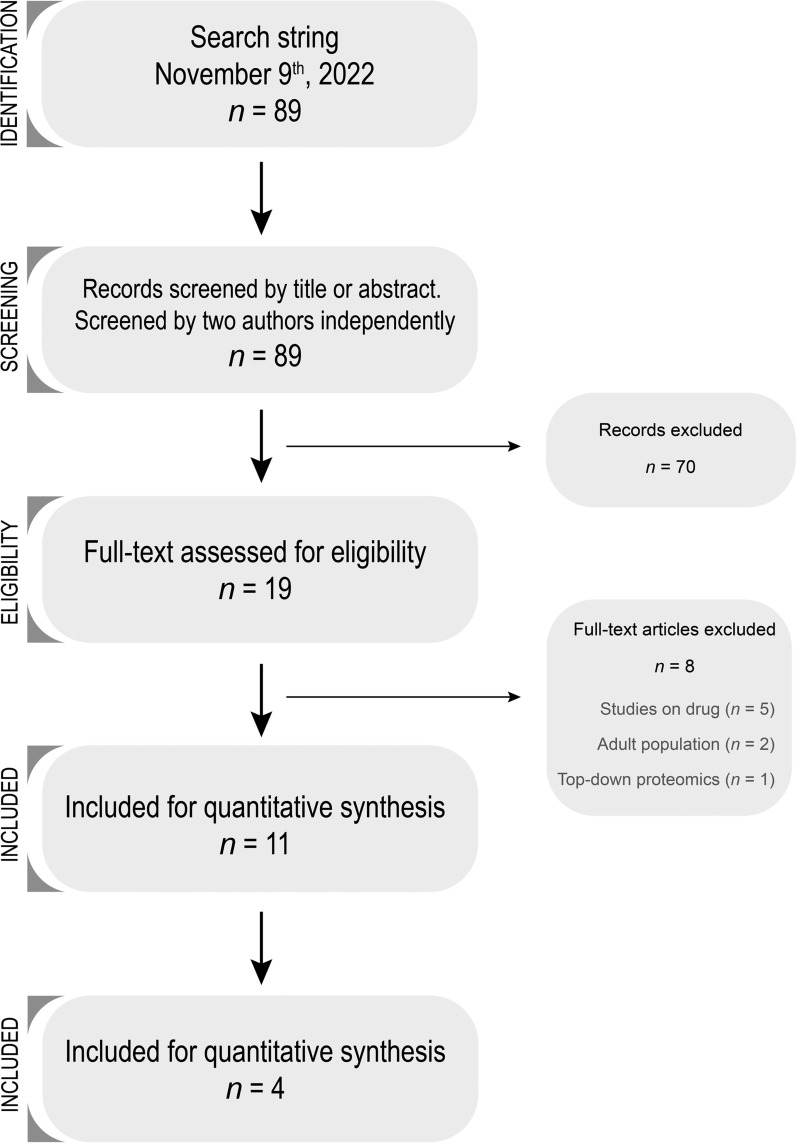
Flow-chart of the search strategy

Figure 1 presents a flow chart summarizing our search strategy. Initially, we identified 89 papers using our search criteria. After reviewing titles and abstracts, we excluded 70 papers for being irrelevant. We then conducted a full-text review of the remaining 19 papers, which led to the exclusion of a further 8 papers. Ultimately, this process resulted in 11 eligible studies. Among these, five studies, including one abstract [25], focused on acute lymphoblastic leukemia (ALL), while the remaining six studies concentrated on primary brain tumors. No studies addressing other pediatric malignancies were found in our search.

### Quality of evidence

In our assessment of the included studies, as per the GRADE guidelines, we have identified them as “very low quality” in terms of evidence, exclusively due to their observational study design. However, as highlighted in Additional file 2: Table S2, it is important to note the unique challenges and limitations inherent in explorative pediatric neuro-oncological research, such as the variability in pre-analytical and analytical workflows and the typically small patient cohorts. These factors, which contribute to heterogeneity and imprecision in results as defined in the GRADE guidelines [18, 19], will persist as challenges in research fields focused on rare cancer diseases.

### Qualitative synthesis

#### Cases and controls

Table 1 provides an overview of the included studies, their respective research aims and included cohort. Discovery cohorts included 161 pediatric patients in total. Hematological malignancies comprised 18 B-ALL, one T-ALL and one T-LL (lymphoblastic lymphoma) patient. The histological origin of primary brain tumors included 82 medulloblastoma, 19 high-grade glioma patients, of which ten had diffuse intrinsic pontine gliomas (DIPG), ten pilocytic astrocytoma, nine grade-II and -III ependymoma, five atypical teratoid rhabdoid tumors (AT/RT), three primitive neuroectodermal tumor (PNET), three germ cell tumor, three gangliocytoma, two choroid plexus carcinoma, two choroid plexus papilloma, two meningiomas, and one hemangioblastoma.

**Table 1:** Overview of included studies

Study, design, year	Malignancy	Research aim	Cohort
Guo et al. Comparative study 2019	CNS B-ALL	CSF-proteome when affected by CNS infiltration of B-ALL	B-ALL (n = 6, CNS positive on “examination”) Healthy controls (n = 6)
Trueworthy et al. Comparative study (poster presentation) 2006	CNS B-ALL	CSF-proteome when affected by CNS infiltration of B-ALL	*(1)* two subgroups of morphologically negative CSF samples on PCR technique of centrifuged cell lysates: First subgroup: MRD negative (n = 3) Second subgroup: MRD positive (n = 3), and *(2)* morphologically positive CSF patients (n = 2)
Fei Mo Comparative study 2019	CNS B-ALL	CSF-proteome before and after ITC + induction therapy of CNS positive B-ALL	B-ALL (n = 6)
Yu et al. Comparative study 2020	CNS B-ALL	Proteomic alterations in CSF induced by chemotherapy	B-ALL (n = 9)
Priola et al. Comparative study 2015	CNS B-ALL CNS T-ALL	Proteomic alterations in CSF correlated with thromboembolic events and induced by chemotherapy	B-ALL (n = 2) T-ALL (n = 2)
Reichl et al. Comparative study 2020	Brain tumor	To quantify CSF-proteome of recurrent medulloblastoma	Recurrent cases of medulloblastoma and prior to treatment (n = 8) Controls (n = 7): age-matched *non*-neoplastic. Not further elaborated
Rajagopal et al. Comparative study 2011	Brain tumor	To investigate putative CSF-proteome biomarkers in medulloblastoma patients	Medulloblastoma (n = 33) Controls (n = 25): age-matched, “leftover samples drawn for other clinical purposes”
de Bont Comparative study 2006	Brain tumor	To detect differences in protein expression profiles of CSF from pediatric patients with and without brain tumors	Medulloblastoma (n = 16); high-grade glioma (n = 7); atypical rhabdoid tumor (n = 2); pilocytic astrocytoma (n = 2); plexus carcinoma (n = 2); anaplastic ependymoma (n = 2); germ cell tumor (n = 1) Control (n = 70; lumbar puncture of pediatric patients 1 year after treatment of ALL [n = 47]; infection [n = 6]; hematological disease [n = 4]; autoimmune disease [n = 2]; idiopathic intracranial hypertension [n = 1]; extra-CNS Hodgkin’s [n = 6]; neuroblastoma [n = 4])
Spreafico et al. Comparative study 2017	Brain tumor	To characterize the CSF proteome of patients with CSF tumors and to identify biomarkers predictive of metastatic sprea	Medulloblastoma (n = 18); grade II and III ependymoma (n = 1 and 4); PNET (n = 2); AT/RT (n = 1); high-grade glioma (n = 1) Controls (n = 13, extra-CNS non-Hodgkin’s lymphoma)
Saratsis et al. Comparative study 2012	Brain tumor	Exploratory study of CSF proteome in pediatric glioma patients	DIPG (n = 10) GBM (n = 1) Age-matched controls (n = 4, not further elaborated)
Bruschi et al. Comparative study 2021	Brain tumor	Putative biomarkers for specific brain tumor subtypes	Pilocytotic astrocytoma (n = 8) Gangliocytoma (n = 3) Medulloblastoma (n = 7), AT/RT (n = 2) PNET (n = 1) *Other* (n = 8) Controls (n = 17, congenital hydrocephalus)

*ALL* acute lymphoblastic leukemia, *CNS* central nervous system, *CSF* cerebrospinal fluid, *ITC* intrathecal chemotherapy, *MRD* Minimal Residual Disease, *PCR* polymerase chain reaction, *DIPG* diffuse intrinsic pontine glioma

Seven of the ten studies included controls in the discovery cohort (*n* = 134), which comprised: “age-matched” controls with no other specification in three studies [26–28], while other studies included healthy controls [29], or controls with other diseases, such as congenital hydrocephalus or other malignancies (extra-CNS Hodgkin’s lymphoma, neuroblastoma and ALL patients 1-year after treatment) [30–32].

Validation cohorts were exclusively utilized in studies related to brain tumors. Three studies used CSF from the discovery cohort to validate findings in Western blots and Sandwich ELISA [27, 30, 31]. One study applied CSF, normal brain tissue, serum and urine as body fluid validation sampled from 22 non-tumor controls, 17 low- or high-grade supratentorial gliomas and nine DIPG [28]. Finally, one study applied the CSF from pediatric patients encompassing 60 various brain tumor entities and 14 extra-CNS non-Hodgkin’s lymphoma [32].

#### Applied protocols and workflow

Additional file 1: Table S1 presents a detailed overview of the various workflows applied in the studies. The CSF samples were collected using three different methods: intra-operatively, by lumbar puncture or through a shunt. The pre-analytical workflow varied, involving centrifugation at speeds ranging from 250xg to 12,000xg for five to ten minutes (these parameters were not reported in two studies [32, 33]). Centrifugation temperature was 4 °C in two protocols, but was not reported in the others. Regarding storage, samples were kept at −20 °C in polypropylene (PP) tubes in one protocol, while they were stored at −80 °C in the others, with the type of tubes not reported). For digesting, tryptic in-solution digestion was used in three protocols, while the remaining eight studies employed two-dimensional gel electrophoresis (2-DE) prior to tryptic digestion.

#### Individual study aims and candidate proteins

Three studies analyzed changes in the CSF proteome during chemotherapy in ALL patients, using repeated sampling from the same individuals [33–35]. In contrast, the other studies collected CSF samples at a single time point and compared these across different groups. Additional file 2: Table S2 presents a detailed list of identified proteins along with their key molecular functions or biological processes. From this, Table 2 summarizes candidate proteins that potentially indicate the presence of malignancy, adverse events, and neuroprotective mechanisms, as derived from our qualitative synthesis.

**Table 2:** Proteins of potential interest

Protein	Measure
Malignancy	
Catalase (increases)	CNS-ALL (1) Occurs in aerobically respiring cells (indicating hypoxic glycolysis) (2) Promotes growth of leukemic cells
Melanoma-derived growth regulatory protein (increases)	Glioma Infrequently expressed in glioma. Expression correlate with lower progression rates
Gamma-enolase (increases)	Brain tumors Known to increase in various tumors
Kallikrein-6 (increases)	Metastasizing/infiltration Degrades extracellular matrix, facilitating ease of infiltration
Adverse events	
Amyloid-like protein 2 (decrease) Plasminogen (decrease) Histidine-rich glycoprotein (decrease)	Thromboemobolism *Amyloid-like protein 2* exert inhibitory effects on coagulations factors *Plasminogen* and *Histidine-rich glycoprotein* deficiencies are prothombotic due to hypofibrinolysis
Apolipoprotein E (increase) Calsyntenin-1 (increase) Glypican-1 (increase) Disintegrin and metalloproteinase domain-containing protein 10 (increase) Spectrin beta chain brain 2 (increase) Prosaposin receptor GPR37 (increase) Sodium/potassium-transporting ATPase subunit alpha-3 (increase) Triosephosphate isomerase (decrease) Progesteron-induced blocking factor 1 (decrease)	Neurotoxicity—involved in pathogenesis of neurodegenerative diseases or neuromuscular dysfinction Alzheimer: *Apolipoprotein E, Calsyntenin-1, Glypican-1, Disintegran metalloproteinase domain-containing protein 10* Parkinsonism: *Ceruloplasmin, Sodium/potassium-transporting ATPase subunit alpha-3* and *Prosaposin receptor GPR37* (juvenile Parkinson) Neuromuscular dysfunction: *Spectrin beta chain brain 2*, *Sodium/potassium-transporting ATPase subunit alpha-3*
Neuromodulin (increase) Gamma-enolase (increase) Prosaposin receptor GPR37 (increase) Prostaglanding-H2 D-isomerase (increase) Immunoglobulin superfamily member 8 (increase) Kallikrein-6 (increase)	Neuroprotective properties (as measure of neurotoxicity)

### Re-analysis of individual patient data

Three studies deposited original mass spectrometry raw files in the PRIDE repository (CNS-ALL *PXD017415* [33]; and brain tumors *PXD022512* [30] and *PXD018226* [26]). One study provided intensity-values obtained from ALL patients, but not the raw mass spectrometry files [29]. In total, the ALL cohort comprised 15 B-ALL patients (six with confirmed CNS involvement and nine with unknown CNS status) and six healthy children. The brain tumor cohort comprised 37 brain tumors (15 Medulloblastoma, eight pilocytic astrocytoma, three Gangliocytoma/Ganglioglioma, two AT/RT, two ependymoma, two germ cell tumors, two meningioma, and one PNET, hemangioblastoma, plexus papilloma, respectively).

### Cohorts analyzed separately (without batch correction)

*The cohorts of ALL*. The Yu cohort consisted of nine B-ALL patients with risk profiles ranging from average to high-risk (age 3.2–12.9 years), with no information of CNS status [33]. Available raw mass spectrometry files were covered all nine patients and measurements at four different treatment stages of each patient: before chemotherapy, during induction, consolidation and maintenance therapy. For our analysis, we exclusively used the raw files obtained before chemotherapy. The Guo cohort included six B-ALL patients with CNS involvement (age 1–11 years) and six “healthy controls”—i.e., children with sterile CSF following examination of encephalitis *obs pro* (age missing) [29].

**Fig. 2:**
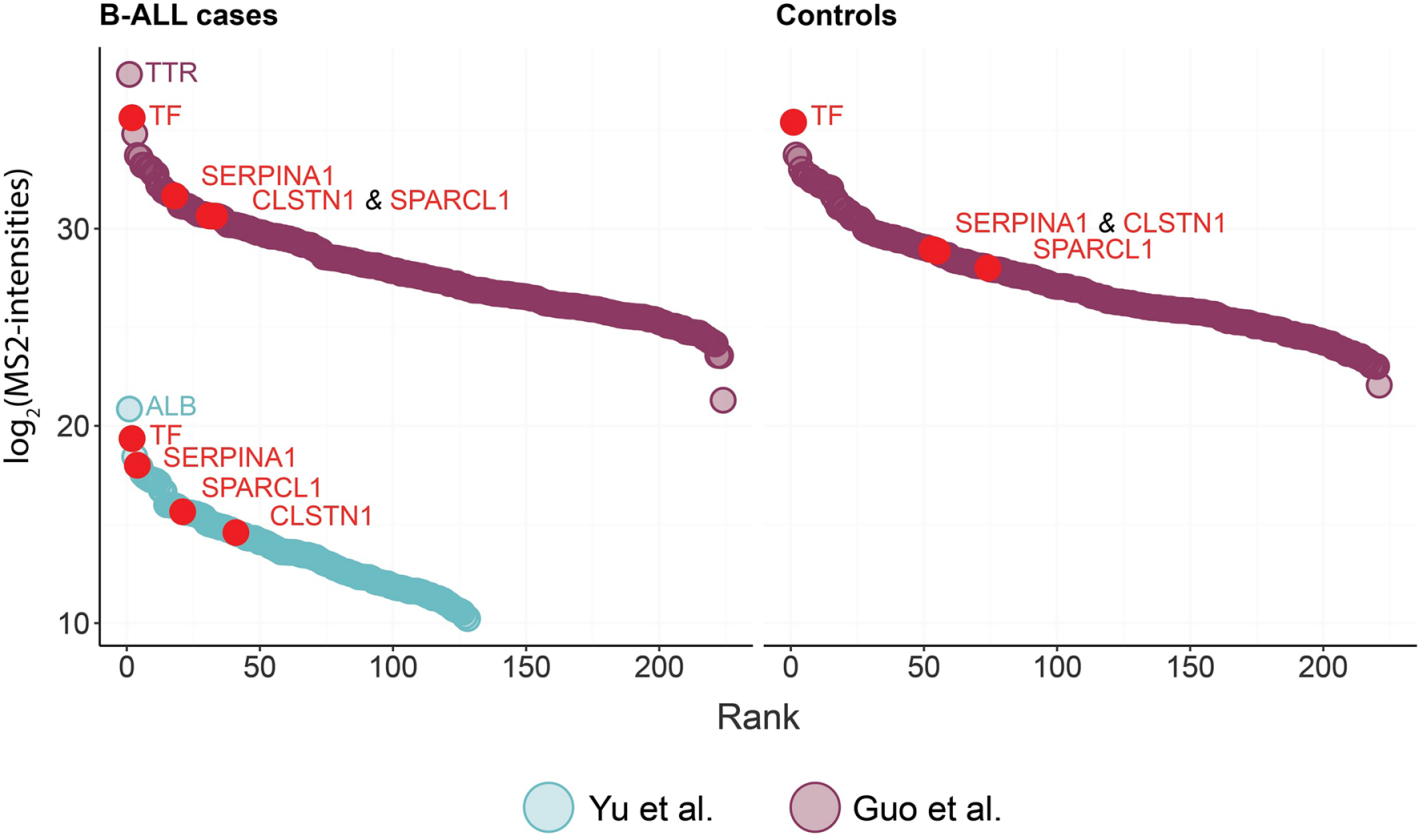
The dynamic range comparing the Yu and Guo cohort

Figure 2 illustrates the dynamic range of cases from both studies and controls from the Guo cohort. The log_2_-intensities obtained from the Guo cohort was considerably higher compared to the Yu cohort.

In total, after excluding potential contaminants, reverse hits and those identified only by site, and requiring valid values in at least 50% of the samples, 237 proteins were uniquely quantified. Specifically, 128 and 224 unique proteins were separately identified in the Yu cohort and Guo cohort. The highest-ranking protein in the Guo cohort was *Transthyretin* (*TTR*) and *Serotransferrin* (*TF*), while *Albumin* (*ALB*) was the highest-ranking protein in the Yu cohort (note that ALB was excluded by Guo et al.). The most significant rank differences between cases and controls in the Guo cohort were observed in *Alpha-1-antitrypsin* (*SERPINA1*) and *SPARC-like protein 1* (*SPARCL1*) (Fig. 2). In the Yu cohort, *SERPINA1* and *SPARCL1* ranked four and 21st out of 128, respectively (note: the Yu cohort has no controls).

*The cohorts of primary brain tumors*. The Reichl cohort comprised eight medulloblastoma patients and seven controls without neoplastic diseases, although further details on these controls were not elaborated [26]. The Bruschi cohort was more diverse, including patients with low-grade gliomas, glioneural tumors, embryonal tumors (among which were seven medulloblastomas), and a varied group of 'Other' entities [30]. In this cohort, patients with congenital hydrocephalus were used as the control group.

A total of 1,159 unique proteins were identified after removing potential contaminants, reverse hits, and those identified only by site. However, this number was reduced to 482 unique proteins when we imposed a criterion of having valid values in at least 50% of both cases and controls.

#### The CSF proteome: medulloblastoma vs controls

**Fig. 3:**
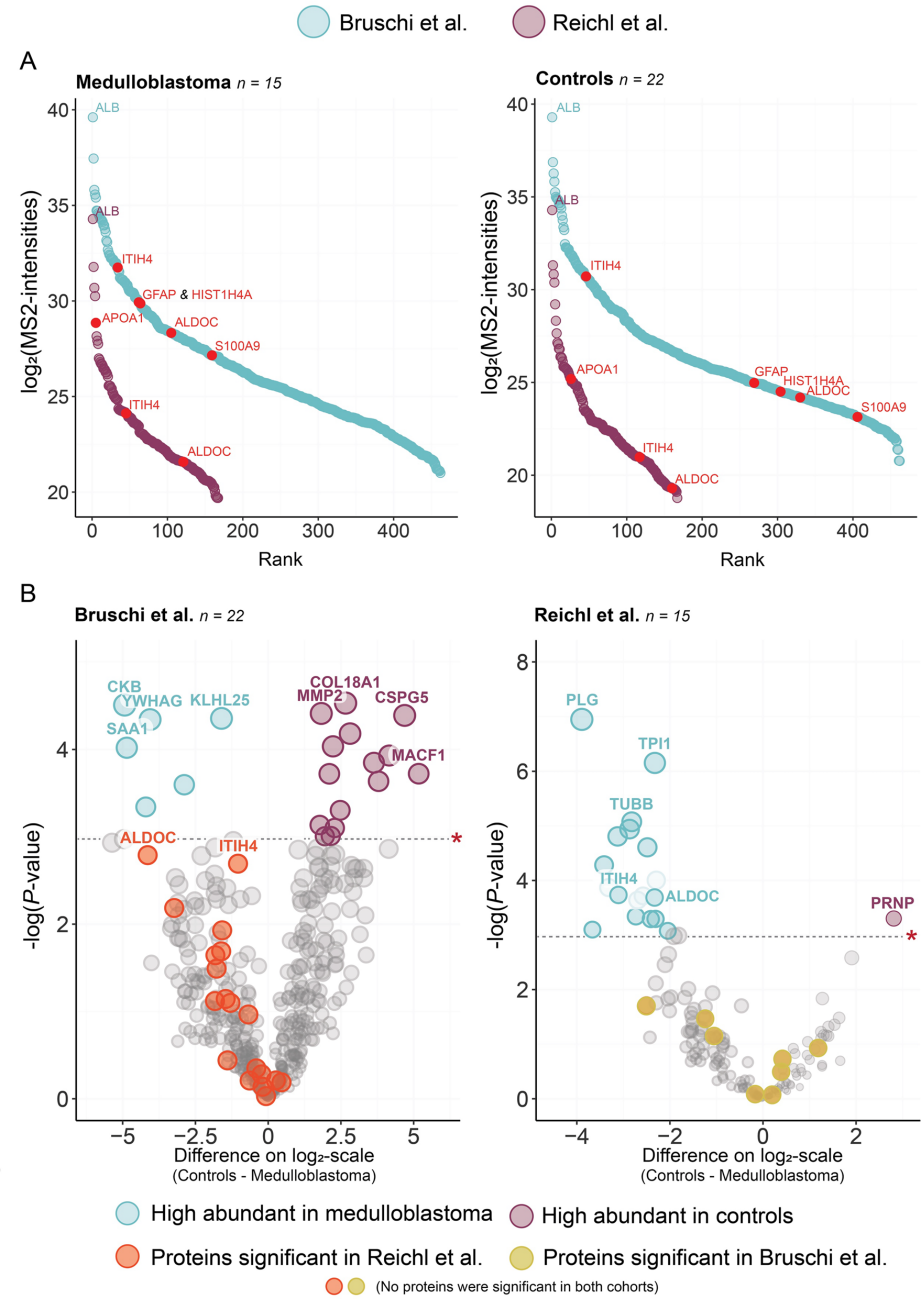
**A** Dynamic ranges for Bruschi and Reichl cohort. **B** Volcano plot based on *t*-test for comparing patients with a medulloblastoma to control. *P-value at 0.05 (uncorrected)

For further analysis, we focused on 15 medulloblastoma cases and 22 controls. There was some inconsistency in how the authors reported the ages of the included participants. In the Bruschi cohort, the median age for medulloblastoma patients was five years (ranging from birth to 15 years), whereas the median age for controls was six years (ranging from 4 to 10 years). In contrast, the Reichl cohort reported an average age of 6.9 years (with a standard deviation of 4.0) for medulloblastoma patients and 9.7 years (standard deviation 5.7) for controls. Figure 3A illustrates the dynamic ranges for medulloblastoma patients and controls in both cohorts, which corresponded to 167 unique proteins in the Reichl cohort and 462 in the Bruschi cohort.

In both cohorts, Albumin (ALB) was the highest-ranking CSF protein for both medulloblastoma patients and controls. However, there was no other consistency in the ranking of proteins between medulloblastoma patients and controls across the two cohorts (Fig. 3A). The most significant differences in CSF protein rank between medulloblastoma patients controls in the Bruschi cohort were *Glial fibrillary acidic protein* (*GFAP)*, *Histone H4* (*HIST1H4A*), *Protein S100-A9* (*S100A9*) and, *Fructose-biphosphate aldolase C* (*ALDOC*). In the Reichl cohort, the proteins with the greatest rank differences were *Apolipoprotein A-I* (*APOA1*) and *Inter-alpha-trypsin inhibitor heavy chain H4* (*ITIH4*) (Fig. 3A).

We used a *t*-test to compare the mean log2-transformed MS2-intensities of each unique protein between medulloblastoma patients and controls. Notably, no same protein showed statistical significance between both cohorts. However, two proteins, *ALDOC* and *ITIH4*, were significantly elevated in medulloblastoma patients within the Reichl cohort. However, these differences were not statistically significant in the Bruschi cohort, but still found to be higher in the CSF proteome of medulloblastoma patients compared to controls (Fig. 3B).

#### Gene ontology enrichment analysis: biological functions

**Fig. 4:**
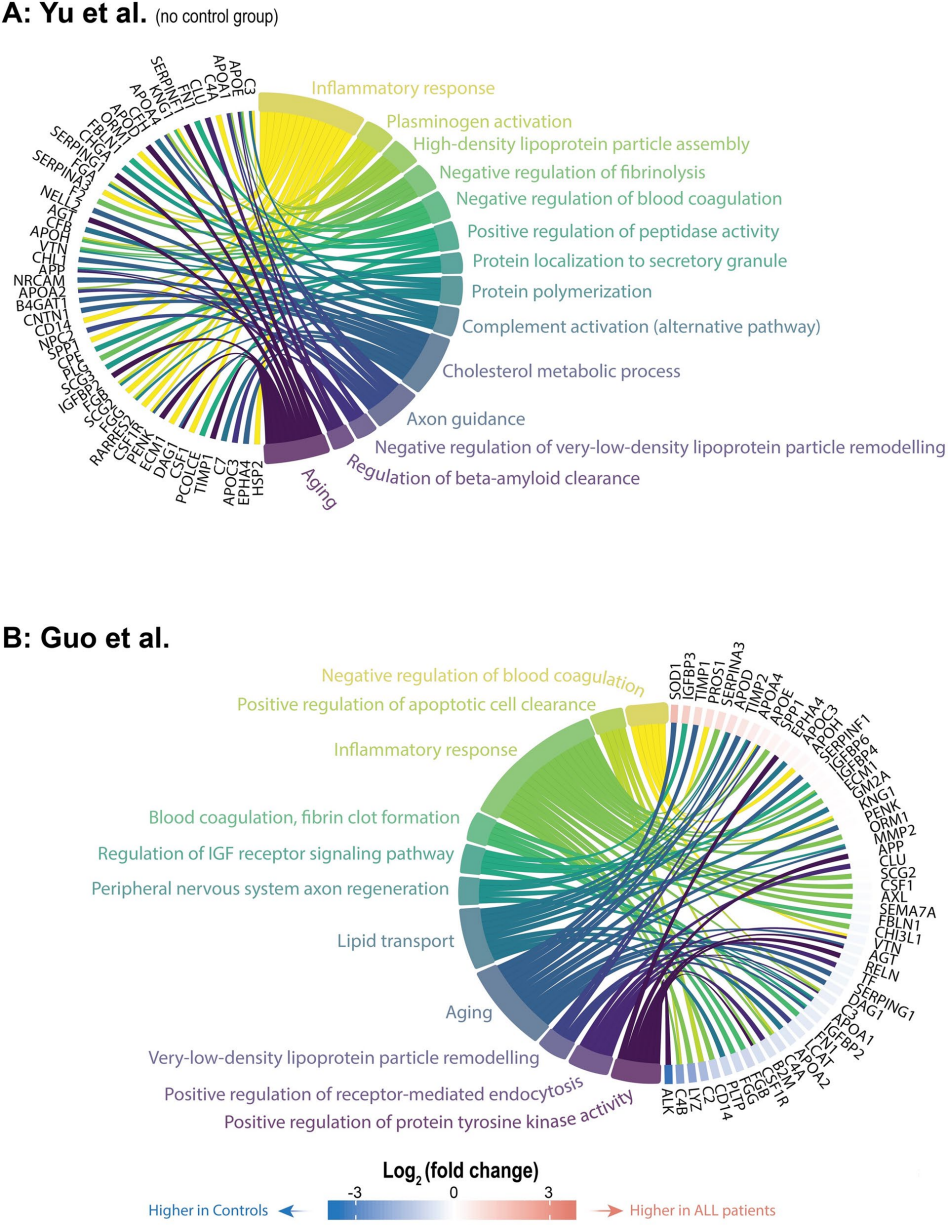
Gene ontology enrichment analysis of proteins expressed in ALL patients in the Yu cohort (**A**, no controls), while comparing ALL patients to controls in the Guo cohort (**B**)

**Fig. 5:**
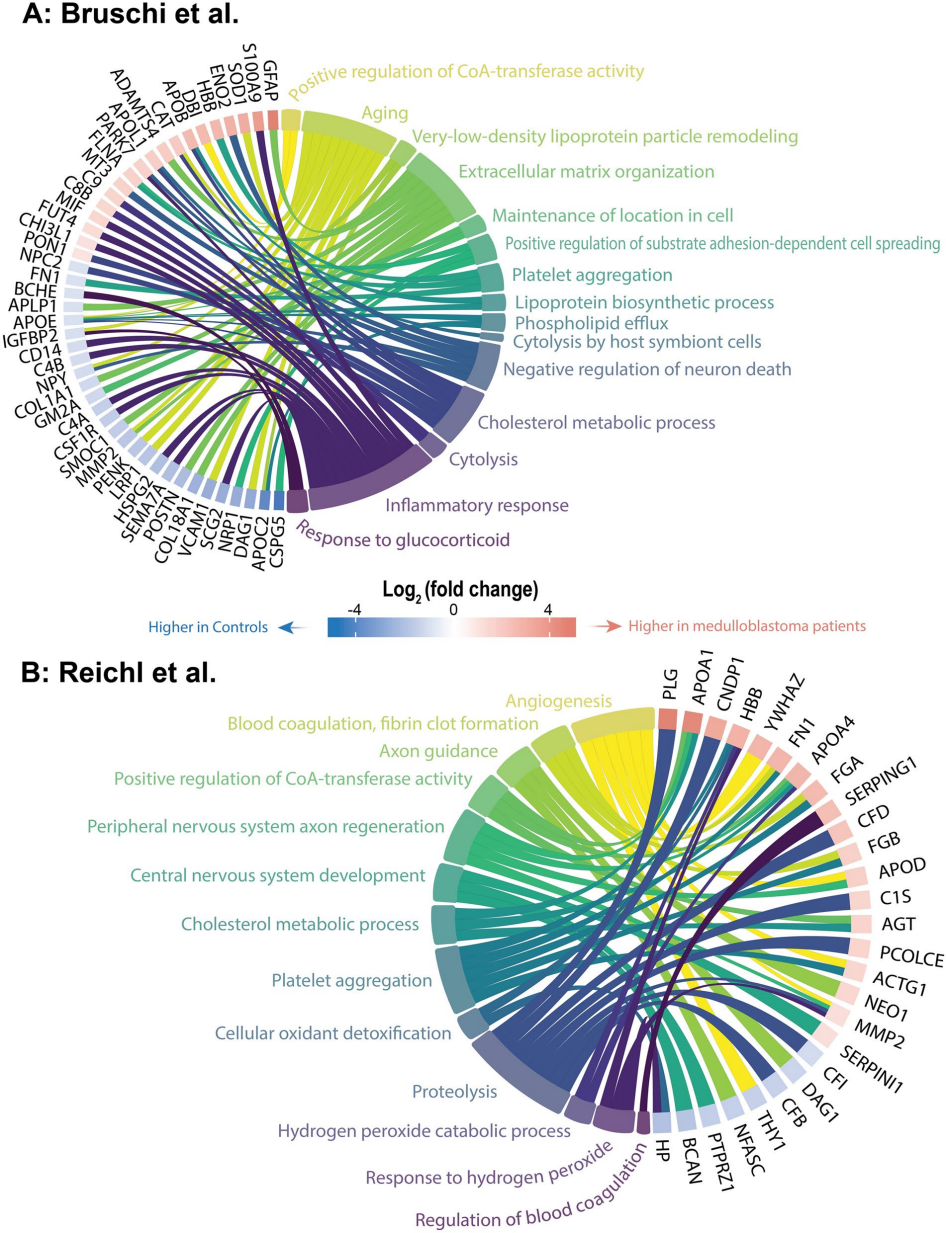
Gene ontology enrichment analysis comparing medulloblastoma patients to controls using the Bruschi (**A**) and Reichl (**B**) cohort

In general, gene ontology (GO) terms analysis indicated common themes in these pediatric cohorts, including *Aging*, *Axon guidance* and, *Central nervous system development* (Figs. 4A, B and 5A, B). Further, the Bruschi cohort showed consistently higher levels of proteins associated with *Aging* Controls compared to medulloblastoma patients (Fig. 5A). This differenve may be attributed to the inclusion of patients with congenital hydrocephalus in the control group. In contrast, no similar GO term difference was observed in the Reichl cohort, which used 'age-matched patients without neoplastic disease' as controls (Fig. 5B). Furthermore, gene ontology enrichment analysis indicated a notable presence of GO terms related to blood contamination across all cohorts. These terms included: *Platelet aggregation*, *Blood coagulation (fibrin clot formation)*, *Plasminogen activation*, *Negative regulation of blood coagulation* and, *Negative regulation of blood coagulation*.

The GO term *Cholesterol metabolic process* exhibited aberrations in three out of the four cohorts (Bruschi et al., Reichl et al., and Yu et al.). Specifically, it was dysregulated in the Yu cohort, which focused on ALL patients; however, this cohort lacked controls and information on CNS status. The GO term was not identified in the Guo cohort (Fig. 4A, B). Contrarily, proteins associating with the GO term were considerably upregulated in medulloblastoma patients when compared with controls in the brain tumor cohorts (Fig. 5A, B). Cholesterol biosynthesis has previously been linked to CNS involvement in ALL, emphasizing its potential relevance [36].

#### Batch correction without imputation: merging of data

**Fig. 6:**
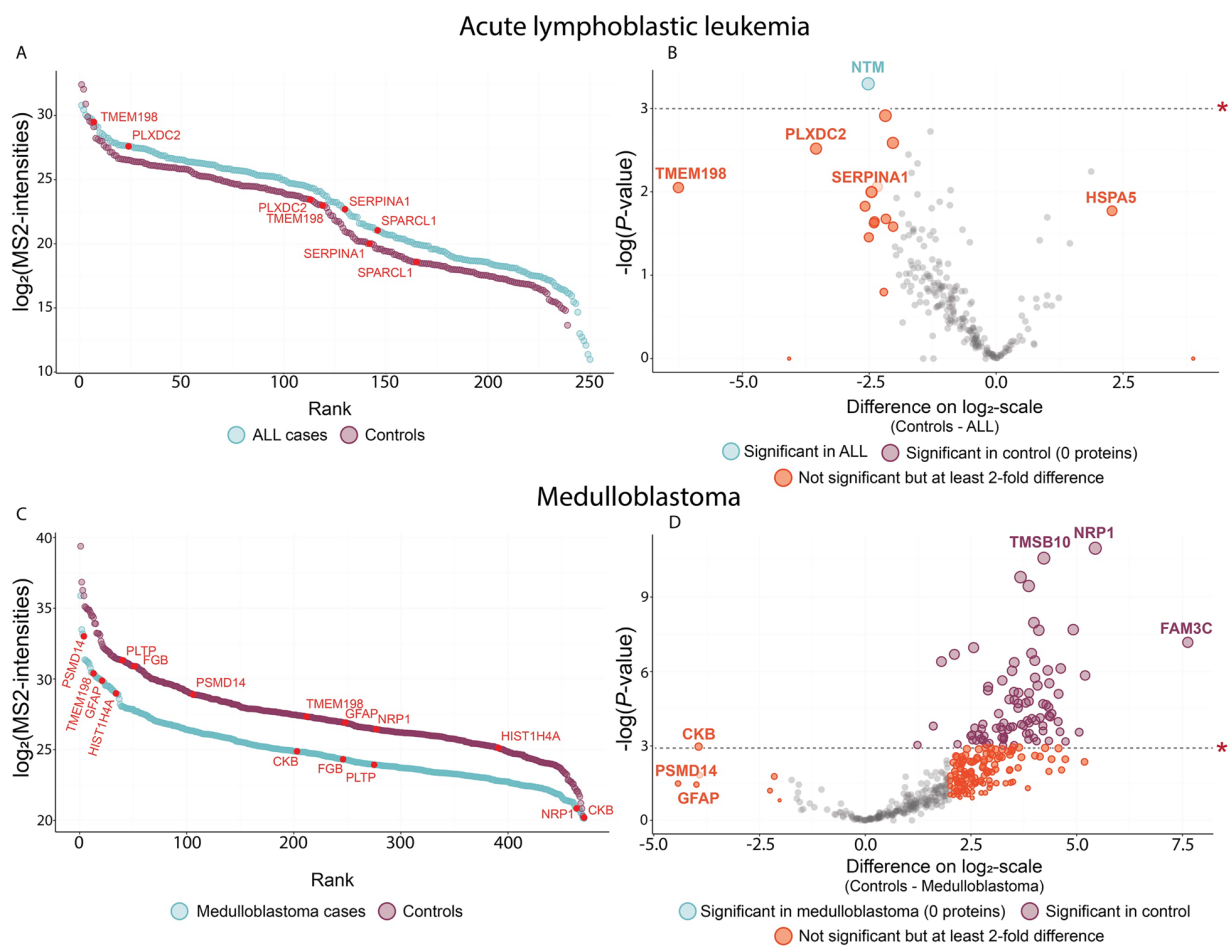
Merging of ALL proteomic data (Yu et al. and Guo et al.) and medulloblastoma proteomic data using the HarmonizR-approach for batch correction without imputation

We applied the recent HarmonizR approach to merge both datasets, encompassing ALL vs. medulloblastoma patients, including controls, without utilizing imputation strategies for missing values. Figure 6 delineates the dynamic ranges and the results of a *t*-test comparing each protein between cases and controls. For ALL patients, the most extreme differences in rank order comprised *Transmembrane Protein 198* (*TMEM198*) and *Plexin domain-containing protein 2* (*PLXDC2*). *SERPINA1* was similarly more abundant in ALL cases with a ~ 5.5-fold increase in MS2-intensities. *SERPINA1* was similarly more abundant in ALL cases with a ~ 5.5-fold increase in MS2-intensities. The only protein with statistically significant difference was *Neurotrimin* (*NTM*), yielding a ~ 5.7-fold increase in mean MS2-intensity in ALL cases (*P* = 0.04) (Fig. 6A, B).

For medulloblastoma, the most significant differences in rank order were observed in *CKB*, *GFAP*, and *26S proteasome non-ATPase regulatory subunit 14* (*PSMD14*). In total, 90 proteins showed statistically significant difference, all of which were less abundant in medulloblastoma patients compared to controls. *Neuropilin-1* (*NRP1*) and *Protein FAM3C* (*FAMC3*) comprised two proteins with high increase in controls (*P* < 0.001) (Fig. 6B, c).

## Discussion

The first outcome was to report and assess the mass spectrometry-based workflows used in in existing literature with an emphasis on the pre-analytical, analytical, and validation processes. A consistent observation throughout our study was the substantial variation in methodologies applied, also in regards to the use of non-tumor controls. Such variation in methodologies complicates the assessment and comparison of existing literature, which is reflected by the GRADE recommendations in terms of quality of evidence.

Specifically, the GRADE recommendations provide a framework for assessing the quality of evidence through consideration of various parameters that influence the synthesis of evidence. Applying the GRADE framework to explorative research, especially in the context of rare cancer diseases such as pediatric CNS cancer, poses some challenges. The GRADE criteria might not fully account for factors that are commonly encountered in such rare diseases, including small sample sizes and variations in pre-analytical and analytical workflows due to rapid technological advancements. This will inevitably lead to a lower quality rating as an inherent consequence of “heterogeneity” and “imprecision”, despite the potential significance of the findings. Moreover, the rarity of these conditions means that standardized research protocols are less established, further complicating the assessment process [37]. It is crucial, therefore, to consider these context-specific challenges when interpreting GRADE assessments in the field of pediatric CNS malignancies. Thus, our practical conclusion was not that the included studies were to be considered as “very low quality” studies, but rather represented opportunities for future advancements in the field. In light of these considerations, we propose several key recommendations to enhance future research in CSF collected from pediatric patients with CNS malignancies. Firstly, standardizing methodologies, particularly in pre-analytical and analytical procedures, is imperative to reduce variability and improve result comparability across studies.

Collaborative efforts should be made to increase sample sizes, possibly through multi-center studies, and bolster statistical robustness. If collaborative efforts are not feasible, ensuring data availability with comprehensive patient characteristics (such as age, specific molecular tumor markers, received treatments, etc.) becomes essential for meaningful comparisons and analyses. Further, comprehensive reporting and open data sharing should be encouraged to facilitate re-analyses and meta-analyses, thereby enriching the field's collective knowledge. Embracing these recommendations will not only overcome the limitations highlighted by the GRADE framework but also foster interdisciplinary collaborations that can bring diverse expertise together, hence offering more innovative approaches to these complex challenges.

Finally, acknowledging the rapid advancements in technology, the adoption of analytical methodologies such as data-independent acquisition (DIA) in mass spectrometry-based proteomics, could enhance the proteome depth and reliability of proteomic analyses.

### Non-tumor controls: cautious use

We derive one cohort-related recommendation from re-analyzing the available data. Here, the gene ontology enrichment analysis suggested that inclusion of non-cancer controls must be considered carefully. Proteome profiling of a patient *vs* controls with an unmatched age may yield proteins that correlate with different developmental stages of CNS. Thus, detection of high- or low-abundant proteins does not necessarily reflect a disease *vs* a disease-free microenvironment, but rather natural and biological processes associated with CNS development. Given the underlying complexity in acquiring CSF from children, it may be tempting to quantitatively include as many samples as possible. However, this should require age matching, age stratification or adjusting to age.

### Cohort comparability and batch correction

We applied the HarmonizR-approach to correct the batch effects and subsequently to compare differences between batch corrected *vs* uncorrected data [22]. However, we encountered a significant challenge with missing values across the studies, which are automatically excluded from the dynamic range and *t*-test analyses, potentially leading to inaccurate estimates. Despite the batch correction, certain proteins, like *SERPINA1* in ALL patients and *CKB* in medulloblastoma patients, showed consistent trends across both the original and corrected data. On the other hand, proteins like NRP1, known to be associated with various pediatric brain tumors and often overexpressed in medulloblastomas [38], rendered counterintuitive estimates in this context. Specifically, the mean MS2-intensity of *NRP1* in controls was about 43 times higher than in medulloblastoma patients, possibly due to the extent incomplete data despite batch correction. While emerging tools like HarmonizR can reduce batch effects, the substantial amount of *missing not at random* data, limited the meaningfulness of merging datasets. Consequently, integrating data across different studies was considered unfeasible.

### Second objective: synthesizing evidence

The second objective focused on synthesizing evidence from previous studies, either through qualitative summaries of findings or quantitative re-analysis of available mass spectrometry data. This process aimed to examine CSF proteome characteristics or biomarkers that could reflect the presence of disease or detect neurotoxicity.

In general, the biological mechanisms facilitating the invasion of malignant cells across the blood–brain-barrier, their dissemination within the CNS and survival in the CSF, are poorly understood [39]. Leukemic blasts and brain tumor cells originate from highly oxygen-rich bone marrow and CNS environments. Still, certain tumor cells can manage to survive in the oxygen-deprived conditions of CSF. Here, previous studies suggest that metabolic adaptability, such as increased fatty acid synthesis, upregulated cholesterol biosynthesis and enhanced glycolysis, might mitigate these cytotoxic effects of CSF, as observed in some pediatric ALL patients [40, 41].

### Quantitative synthesis: individual patient-data analysis

As elaborated, merging and subsequent batch correction of data proved unfeasible, even within the same disease entities. As a result, we compared the datasets separately using rank orders and t-tests. The comparison was further complicated by the unknown CNS status in the Yu cohort, which affected the analysis of CNS-ALL, CNS-naïve ALL patients, and controls. The most pronounced differences in protein rank between cases and controls were observed for SERPINA1 and SPARCL1 in the Guo cohort. In the Yu cohort, these proteins were also identified, ranking 4th and 21st out of 128, respectively. Overexpression of *SERPINA1* is linked to a poor prognosis in various tumors, including colorectal cancer, breast cancer and non-small cell lung cancer, but has not been described for ALL [42–44]. SPARCL1, known as a tumor suppressor, is associated with poorer survival in several cancers when downregulated.

In the studies on brain tumor patients, the dynamic range of MS2 intensities between medulloblastoma patients and controls varied significantly when comparing the two cohorts. However, *ITIH4* was found to be more abundant in medulloblastoma patients compared to controls, a finding also reported in another study included in the qualitative synthesis [32]. In addition, *ITIH4* is known to be associated with other cancers like hepatocellular carcinoma and gastric cancer [45, 46]. *ALDOC*, an astrocyte-specific marker in all regions of the brain, is linked to various gliomas, but its presence in medulloblastoma has not been reported. *GFAP,* also expressed in astrocytes, has an uncertain role in medulloblastoma, although some studies suggest its expression in medulloblastoma cells [47–49].

### Corroboration of previous findings

The gene ontology enrichment analysis indicated that *Cholesterol metabolic process* was a highly upregulated biological function in CSF from two (three if counting Yu et al. without a non-cancer control) of four studies. It was recently found that upregulation of cholesterol biosynthetic pathways were linked to CNS-ALL [36]. In the CNS, however, cholesterol exist as the 24(S)-hydroxycholesterol isoform, which is only produced in CNS. This bioavailability of this isoform in CSF is scarce, and is transported within CNS in the form of *Apolipoprotein E (APOE)*-containing lipoprotein particles secreted mainly by glial cells [50]. Most of the proteins that associated with *Cholesterol metabolic process* in the gene ontology enrichment analysis herein are related to systemic transportation of cholesterol, which could suggest blood contamination. We did not identify any of proteins associated with the biosynthetic pathways of cholesterol previously reported as upregulated in patients with isolated CNS relapse [36].

### Qualitative synthesis

Proteins with neurotrophic and neuroprotective properties were notably upregulated in the CSF of brain tumor patients [26, 27, 32]. This group included *Neuromodulin* (*GAP43*) involved in nerve growth, *Gamma-enolase* (*ENO2*) promoting neuron survival (which has been known to increase in brain tumor patients [51, 52]), *Prosaposin receptor GPR37* (*GPR37*) and *Prostaglandin-H2 D-isomerase* (*PTGDS*) with anti-apoptopic effects oligodendrocytes [53–56], and *Immunoglobulin superfamily member 8* (*IGSF8*) involved in neurite outgrowth and maintenance [57]. These findings may reflect both chemotherapy-induced apoptosis in malignant cells and protective responses in normal brain tissue.

#### The CSF proteome and biomarkers of CNS malignancy

Certain CSF proteins like the *Probable ATP-dependent RNA helicase* (*DDX41*), detected during chemotherapy in some ALL patients, may indicate specific malignancies [35]. *DDX41* mutations are linked to cancer predisposition syndrome characterized by increased susceptibility to hematological malignancies [58–60]. *Melanoma-derived growth regulatory protein* (*MIA*), infrequently expressed in gliomas, can signal slower progression in high-grade glioma [61]. More general markers of malignancy include *Catalase* (*CAT*), which was detected in the CSF of B- and T-ALL patients with cytospin CNS2-status [35], supporting adaption to hypoxic glycolysis in CNS-ALL blasts [40]. Further, the serine protease *Kallikrein-6* (*KLK6*) facilitate tumor invasion by degrading the extracellular matrix [62]. In this context, *KLK6* was specifically upregulated in CSF of CNS-ALL patients (*n* = 6 with cytospin CNS2 or CNS3-status during induction therapy) [34].

#### The CSF proteome and biomarkers of adverse events during chemotherapy

Alterations in the CSF proteome during chemotherapy may indicate early stages of neurotoxicity and thromboembolic events. Chemotherapy drugs like methotrexate and cytarabine can alter CSF composition, potentially leading to neurological deficits [63]. For example, permanent neurological deficits affect up to 14% of pediatric ALL patients receiving intrathecal methotrexate [64–66]. Herein, three studies included repeated CSF sampling to investigate alterations in the proteome during treatment of ALL with the beforementioned chemotherapy. A total of 18 B-ALL, one T-ALL and one T-LL patients were included, of which seven were reported with CNS-ALL based on cytospin diagnostics [33–35]. Studies have shown CSF proteome changes similar to Alzheimer's disease during chemotherapy, including increases in *Apolipoprotein E* (*APOE*) and *Calsyntenin-1* (*CLSTN1*) [67–69], and decreases in *Sodium/potassium-transporting ATPase subunit alpha-3* (*ATP1A3*), linked to several neurological disorders [70–75].

Prothrombotic changes, such as decreases in *Fibrinogen alpha chain* (*FGA*) and *Fibrinogen gamma chain* (*FGG*), suggest a risk of thromboembolic complications [76–78]. *Plasminogen* (*PLG*) deficiency is associated with susceptibility to thrombosis [79, 80]. Decreased PLG levels, observed in a B-ALL patient experiencing a central vein thrombosis after Pegasparginase treatment, may support this risk [35].

### In summary

In this study, we pursued two primary objectives: first, to assess mass spectrometry-based workflows detailed in existing literature concerning pre-analytical, analytical, and validation processes in pediatric CNS malignancies, and synthesizing evidence from previous CSF proteomics research, through qualitative and quantitative methods. We found significant methodological variability and challenges in using non-tumor controls, impacting the assessment and comparison of studies as per the GRADE criteria for quality of evidence. This underscores the need to standardize methodologies to enhance comparability in future research. Our gene ontology enrichment analysis emphasized the necessity for careful selection of controls, particularly considering age differences that could influence protein profile interpretations as age disparities could may cause misleading protein correlations more reflective of natural CNS developmental stages rather than disease states. Despite attempts to merge and batch-correct data, the extensive missing values limited the feasibility of integrating data from different studies, highlighting the need for improved data sharing and harmonization.

### Supplementary Information

**Additional file 1: Table S1.** Applied workflow for mass spectrometry proteome analysis.

**Additional file 2: Table S2.** Aggregated list of potential CSF biomarkers from the reviewed articles.

## Data Availability

Re-analysis of available raw mass spectrometry files were downloaded from the following repositories: *PXD017415*, *PXD022512* and *PXD018226.*
