# Supplementary material for: Mass spectrometry-based proteomics of cerebrospinal fluid in pediatric central nervous system malignancies: a systematic review with meta-analysis of individual patient data
Source: Fluids Barriers CNS. 2024 Feb 13;21:14. doi: 10.1186/s12987-024-00515-x (PMC10863112; doi:10.1186/s12987-024-00515-x)
Supplement: Supplementary file 1 — Additional file 1: Table S1. Applied workflow for mass spectrometry proteome analysis. [file 12987_2024_515_MOESM1_ESM.docx]

**Additional Table 1.** Applied workflow for mass spectrometry proteome analysis.

|  | Pre-analytical | | | Analytical | | | Validation | | |
| --- | --- | --- | --- | --- | --- | --- | --- | --- | --- |
| Study | **CSF collection** | **Before storage** | **Storage before analysis** | **Amount of CSF** | **Discovery workflow** | **Proteome depth** | **Cohort** | **Analytical technique** | **Data availability** |
|  | **Acute lymphoblastic leukemia** | | | | | | | | |
| Guo et al.  (2019) | Method & volume not stated | Cent. 5 min @ 3,000 xg at 4^o^C | -80 ^o^C.  No tube info. | 30 µL | Tryptic digestion of proteins. Peptide analysis by nLC-MSMS, 60 min gradient, Top20 DDA method.  Instrument: Easy-nLC 1000 & Q-Exactive Plus/Q-Exactive.  Software for protein ID and quantification: MaxQuant v1.3 Protein sequence database: SwissProt (release: Unknown). | 455 non-redundant proteins identified (315 protein groups)  51 proteins were differentially expresed (32 up, 19 down) | NA | NA | No |
| Trueworthy et al.  (2006) | NA | NA | NA | NA | NA | NA | NA | NA | No |
| Mo et al  (2019) | Lumbar puncture.  Volume not stated. | Cent. 10 min @ 3,000 rpm (xg not stated) at 4^o^C | -80 ^o^C.  No tube info. | 30 µL | Tryptic digestion of proteins. Peptide analysis by nLC-MSMS, 60 min gradient, Top20 DDA method.  Instrument: Easy-nLC 1000 & Q-Exactive Plus/Q-Exactive.  Software for protein ID and quantification: MaxQuant v1.3 Protein sequence database: SwissProt (release: Unknown). | 428 proteins identified | NA | NA | No |
| Yu et al.  (2020) | Lumbar puncture (1-2 mL) | Cent. no info / not performed.  (91 % of samples had low RBC counts < 1/uL,  9 % of samples had RBC counts in the interval 124-4550 cells/uL) | NA | Amount: 30 ug protein per sample | Tryptic digestion of proteins followed by DiLeu labelling (4-plex) of desalted peptides.  Peptide analysis by nLC-MSMS,  90 min gradient, Top20 DDA method  Instrument: Dionex Ultimate & Q-Exactive HF  Software for protein ID: Proteome Discoverer v 2.1  Protein sequence database: UniProt with 20,152 entries (Aug 2016) | 341 proteins identified. Among these 258 were quantified at all timepoints in at least 5 pts.  51 proteins (wk5), 21 proteins (wk 10-14) and 17 proteins (wk 24-28) were reported differentially expressed relative to wk 1.   63 proteins were found to have significant alterations during the experiment (ANOVA analysis). | NA | NA | ProteomeXchange identifier: PXD017415 |
| Priola et al.  (2015) | Lumbar puncture (1 mL) | Cent. 10 min @ 1,500 rpm (xg not stated). Temp. not stated.  Frozen within 1 hr from time of collection | -80 ^o^C.  No tube info. | 450-760 µL CSF containing 60-308 µg protein | Top 14 depletion of most abundant proteins (MARS-14). Tryptic digestion of proteins. Peptide analysis (1 ug/inj) by nLC-HDMS^E^, 90 min gradient. Instrument: AcquityUPLC & Synapt G2. Software for LC alignment:  Rosetta Elucidator v3.3 Software for protein ID and quantification: ProteinLynx Global Server v2.5.2, Protein sequence database: Customized SwissProt (release: Unknown) with reverse entries | 635 proteins (406 proteins with 2 or more peptides).    Reanalysis of 3 of 4 samples and inclusion of one new pt. sample (with thrombosis):  646 proteins (410 proteins with 2 or more peptides), | NA | NA | No |
|  | **Brain tumors** | | | | | | | | |
| Reichl et al.  (2020) | Ommaya resevoir | Frozen immediately | -80 ^o^C.  No tube info. | 500 µL CSF | Protein from CSF was precipitated using ethanol precipitation  20 ug protein were then loaded onto a 10 kDa cut-off spinfilter and digested using trypsin by the FASP method  Peptide analysis by nLC-MSMS,  85 min gradient, Top8 DDA method  Instrument: Dionex Ultimate 3000 system coupled to a Q-Exactive  Software for protein ID and quantification: MaxQuant v1.6.1.0  Protein sequence database: SwissProt (Ver 03/2018, 20316 sequences) | 729 proteins were identified.  178 proteins were found significantly regulated | NA | NA | ProteomeXchange identifiers: PXD018226, 10.6019/PXD018226 |
| Rajagopal et al.  (2011) | Ventricular (at tumor resection); or lumbar puncture (10-14 post-op.); or shunt. Volumes not stated. | Cent. 10 min @ 3000 xg.  Temp. not stated. | -20 °C in PP tubes | 75 µg protein per sample | 2D-GE (cCBB staining). In-gel digestion of spots of interests. Protein ID by MALDI-TOF/TOF  (ABI 4700). Database searched: SwissProt (release: Unknown). | 160 protein spots detected per gel,  76 spots identified representing 25 proteins | Validation cohort same as discovery cohort | PGD2S: Sandwich ELISA ApoE: No validation, Clusterin: No validation | No |
| de Bont et al.  (2006) | Lumbar puncture. Volumes not stated. | Cent. 5 min @ 250 xg.Temp. not stated. | -80 ^o^C.  No tube info. | 2x 5 µg CSF-protein. | Strong anion-exchange chromatography surface based SELDI-TOF mass spectrometry,  Instrument: Q10 ProteinChips scanned in a PBS IIc instrument in positive mode,  Software for data analysis: ProteinChip Biomarker Wizard, v 3.1  Potential marker candidate was purified and then identified by peptide mass fingerprinting | 247 protein peaks of unknown origin including 123 peaks showing differential expression. One protein identified. | Validation cohort same as discovery cohort | Western blot with 3 pt and 3 ctrl samples probing for Apo A-II,  IHC probing for Apo A-II in brain tissue. No specific cellular staining observed. | No |
| Spreafico et al.  (2017) | Lumbar puncture. Volumes not stated. | Cent. not stated. Temp. not stated. | -80 ^o^C.  No tube info. | 500 µL CSF | Cent. 7 min @ 16,100 xg @ 4°C after thaw.  CSF incubated with hydrogel nanoparticles to depleat for high Mr proteins followed by tryptic digestion of depleated samples Peptide analysis by LC-MSMS, 120 min gradient, Top5 DDA method. Instrument: LTQ-Orbitrap  Software for peptide/protein ID: SEQUEST (ver. not stated). Protein sequence database: NCBI (release not stated). Software for MS1 based protein quant.: BioSieve (ver. not stated). Software for MS2 (spectral count) based protein quant.:  Scaffold (ver. not stated). | In total 558 proteins were identified.  On average 68 ± 34 (stdev) proteins were detected per sample. | CSF samples: Pts (Diag with brain tumor, n=60), Ctrls (Diag with extra-CNS non-Hodgkin’s lymphoma, n=14)   Samples from the discovery cohort were also included in validation experiments by WB and RPPA | Proteins evaluated by western blot (blots not shown):  IGSF8, ITIH4, PCOLCE, FGA, FGG, GFRa2, COL18A1, COL1A2, HRG, IGFBP4, MGP, NPDC1, RNaseA, Selenoprotein P.  Proteins evaluated by RPPA: COL18A1, GFRa2, ITIH4, PCOLCE, HRG, MGP, NPDC1, RNaseA.  Proteins evaluated by ELISA: COL18A1, COL1A1, IGFBP4, IGSF8, PCOLCE, Selenoprotein P | No |
| Saratsis et al.  (2012) | CSF collected from patients with brain tumors intra-operatively, lumbar puncture or post-mortem.  Volume not stated. | Cent. 10 min @ 12,000 xg. Temp. 4 °C | NA | Amount: 110 µg protein per sample | Protein from CSF was concentrated using 3 kDa cut-off spin filters, separated by SDS-PAGE gels, visualized by cCBB staining and converted into peptides by in-gel digestion.  Peptide analysis by nLC-MSMS, (gradient length not stated), Top5 DDA method  Instrument: nLC (Agilent) & Orbitrap XL  Software for protein ID: Sequest run via the Bioworks Browser  Protein sequence database: UniProt (Nov 2010).  Protein quantitation: Spectral counting using ProteoIQ software followed by analysis in Partek Genomics Suite | 528 proteins identified.   73 proteins were upregulated (fold change ≥ 2) in 3 or more of the DIPG CSF samples relative to ctrls  157 proteins were downregulated (fold change ≤ 2) in 3 or more of the DIPG CSF samples relative to ctrls | Validation in CSF, brain tissue, serum and urine:  **CSF** **Pts.(n=26)** -DIPG (n= 9) -Low and high grade supratentorial glioma (n=17) **Ctrls (n= 22)**  **Brain tissue Pts (n=12):** -DIPG (n=10) -STG (n=2)  **Serum** -DIPG (n=2) -Ctrls (n=3)  **Urine** -DIPG (n=3) -Ctrls (n=3) | Proteins evaluated by WB analysis of CSF, serum and urine: CypA and DDAH1  IHC analysis of tissue collected from tumor and from healthy tissue (Brain stem or frontal lope) | No |
| Bruschi et al.  (2021) | Extraventricular drainage  Volume not stated. | Cent. 10 min @ 3000 xg.  Temp. not stated | -80 ^o^C.  No tube info. | 25 µg protein per sample (measured after precipitation with sodium deoxycholate/trichloroacetic acid) | Tryptic digestion of proteins,  Peptide analysis by nLC-MSMS,  150 min gradient, TopN method with ion-intensity based decision-tree for fragmentation by CID/IT or HCD/FT  Instrument: Dionex Ultimate 3000 coupled to an Orbitrap Fusion Tribrid  Software for protein ID and quantification: MaxQuant v1.6.2.6  Protein sequence database: UniProt (release UP000005640_9606, Apr 2018) | 1789 proteins were identified including 1335 proteins identified in both control and tumor samples and 263 proteins observed solely in control samples and 191 proteins observed only in tumor samples.   741 proteins were quantified in at least 70 % of all analysed samples.  241 proteins were found differentially expressed with 228 proteins enriched in control CSF and 13 proteins in tumor CSF. | Same cohort as discovery cohort supplemented with:  **Embryonal tumors (n=1)** -Atypical teratoid rhabdoid tumor (n=1)  **Other brain tumor (n=1)**  **Controls (n=20):** -Congenital hydrocephalus (n=5) -Post-hemorrhagic (n=15) | Proteins evaluated by ELISA: TAF15, S100B, TMSB4X, CD109, 14.3.3g, HSP90 alpha | ProteomeXchange identifier: PXD022512 |
