# Supplementary material for: Mass spectrometry-based proteomics of cerebrospinal fluid in pediatric central nervous system malignancies: a systematic review with meta-analysis of individual patient data
Source: Fluids Barriers CNS. 2024 Feb 13;21:14. doi: 10.1186/s12987-024-00515-x (PMC10863112; doi:10.1186/s12987-024-00515-x)
Supplement: Supplementary file 2 — Additional file 2: Table S2. Aggregated list of potential CSF biomarkers from the reviewed articles. [file 12987_2024_515_MOESM2_ESM.docx]

**Additional Table 2**. Aggregated list of potential CSF biomarkers from the reviewed articles.

| Study, design, year | Research aim and cohort | Findings | Molecular function or  Biological process summarized | Involvement in disease |
| --- | --- | --- | --- | --- |
| Acute lymphoblastic leukemia: central nervous system infiltration | | | | |
| Guo et al.  Comparative study  (2019) | **Aim**  CSF-proteome in CNS infiltration of B-ALL  **Cohort**  ALL (n=6, CNS positive on “examination”)  Healthy controls (n=6) | Two hierarchical clusters encompassing ten core proteins based on significantly different expressed CSF-proteomes (paired t-test).  **Cluster 1**, upregulated proteins in the CSF-proteome of ALL-CNS:  *TIMP Metallopeptidase Inhibitor 1*, *Complement factor I*, *Fibronectin 1*.  **Cluster 2**, downregulated proteins in the CSF-proteome of ALL-CNS:  *Galectin-3-binding protein*, *Alpha-2-Macroglobulin*, *Alpha 2-HS Glycoprotein*, *Histidine-rich glycoprotein*, *Inter-Alpha-Trypsin Inhibitor Heavy Chain 4*, *Complement C2*, and *Complement C4a*. | **Upregulated**  Growth factor, protease inhibitor, innate immune response, acute-phase, angiogenesis  **Downregulated**  Cell adhesion, protease inhibitor, mineral balance, angiogenesis, blood coagulation, fibrinolysis, acute-phase, innate immune respons, inflammatory response | *Alpha 2-HS-Glycoprotein* 🡪 Alopecia-mental retardation syndrome 1  *Histidine-rich glycoprotein* (deficiency) 🡪 thrombophilia |
| Trueworthy et al.  Comparative study (poster presentation)  (2006) | **Aim**  CSF-proteome in CNS infiltration of B-ALL  **Cohort**  *(1)* two subgroups of morphologically negative CSF samples on PCR technique of centrifuged cell lysates:  First subgroup: MRD negative (n=3)  Second subgroup: MRD positive (n=3), and  *(2)* morphologically positive CSF patients (n=2) | It was not specified what method were applied to test significant difference.  Ad *(1)*  CSF-proteome of MRD positive in reference to MRD negative.  **Upregulated**: *Zinc Finger Protein, Bromodomain adjacent to Zinc Finger Domain, Sphingomyelin Phosphodiesterase 3, Osteopontin*, *immunoglobins* (*kappa, Lamda heavy chain*) and *Major Histocompatibility Complex Class II*  **Downregulated**: *Amyloid-beta precursor protein* and *Cystatin C*.  Ad *(2)*  The morphologically positive CSF patients (n=2) confirmed four and one of up- *versus* downregulated protein(s), respectively. | **Upregulated**  DNA-binding, transcription, cell cycle, cell adhesion, immune response  **Downregulated**  Apoptosis, cell adhesion, protease inhibitor, amyloid beta-binding | *Amyloid-beta precursor protein* 🡪   - Alzheimer disease 1 (familial early-onset form) - Cerebral amyloid angiopathy, APP-related   *Cystatin C* 🡪   - amyloidosis 6 (Cystatin C amyloid accumulates in the walls of arteries, arterioles, and sometimes capillaries and veins of the brain) - Age-related macular degeneration - Potential CSF biomarker for Creutzfeldt-Jakob disease |
| Acute lymphoblastic leukemia: serial CSF-proteome samples during chemotherapy | | | | |
| Fei Mo  Comparative study  (2019) | **Aim**  CSF-proteome before and after ITC + induction therapy of CNS positive B-ALL  Time duration not reported.  **Cohort**  B-ALL (n=6)  Treatment protocol: CCCG-2015  ITC: Methotrexate, cytarabine and dexamethasone.  Induction: Prednisolone, daunorubicin, vincristine and pegaspargase. | Significantly different expressed CSF-proteomes (paired t-test, p<0.05) between before and after induction therapy.  **Upregulated**: *Complement C4A, Histidine-rich glycoprotein*, *Apolipoprotein A1, Kallikrein-6,* C*arnosine dipeptidase 1, Complement factor H* and *Alpha-2-Macroglobulin*.  **Downregulated**: *Apolipoprotein D*, *WFIKKN2* (*WAP, follistatin/kazal, immunoglobulin, kunitz and netrin domain containing 2*) and, *Osteonectin* | **Upregulated**  Innate immune response, blood coagulation, cholesterol/lipid metabolism, serine protease inhibitor  **Downregulated**  Lipid-binding, protease inhibitor, metalloenzyme inhibitor | *Histidine-rich glycoprotein* (deficiency) 🡪 thrombophilia  *Apolipoprotein A1* (deficiency) 🡪   - Primary hypoalphalipoproteinemia - Amyloidosis 8 (no CNS involvement) |
| Yu et al  Comparative study  (2020) | **Aim**  Proteomic alterations in CSF induced by chemotherapy.  **Cohort**  B-ALL (n=9)  Treatment protocol: AALL1131  ITC: Methotrexate and cytarabine.  Chemotherapy: cytarabine, vincristine, dexamethasone, doxorubicine, pegaspargase, methotrexate, mercaptopurine, cyclophosphamide, thioguanine.  Samples collected at week 1 (before treatment), week 5 (after induction), week 10-14 (consolidation phase) and week 24-28 (maintenance phase). | In total, 63 proteins exhibited significant expression (ANOVA, p<0.1). There were two hierarchical clusters encompassing week(s) 5, 10 – 14 and 24 – 28 compiled and compared to week 1, i.e. all samples during entire treatment compared to before treatment.  **Cluster 1** upregulated proteins during chemotherapy: *Apolipoprotein E, Clusterin, Zinc-alpha-2-glycoprotein, Contactin 1, Immunoglobulin Heavy Variable 3-23, Calsyntenin-1, Cadherin-2, Type I collagen, Complement C1s, Neural cell adhesion molecule 2, Collagen alpha-1(VI) chain, Apolipoprotein A-IV* and, *Complement C7*.  **Cluster 2** downregulated proteins during chemotherapy*: Immunoglobulin Kappa Variable 1-5,* *Vitronectin,* *Amyloid-like protein 2, Complement C1q subcomponent subunit C, Amyloid-beta precursor protein, Alpha-1-acid glycoprotein 2, Inter-Alpha-Trypsin Inhibitor Heavy Chain 4, Immunoglobulin Lambda Constant 2, Immunoglobulin heavy constant gamma 3, Galectin-3-binding protein, Fibrinogen gamma chain,* *Serum amyloid A-1, Fibrinogen alpha chain,* *Complement factor B*, *Alpha-2-antiplasmin, Immunoglobulin lambda-like polypeptide 5* and, *Tetranectin*. | **Upregulated**  Cholesterol/lipid metabolism, innate and adaptive immune response, lipid degeneration, cell adhesion, lipid transport  **Downregulated**  Protease inhibitor, cell adhesion, Heparin-binding, acute phase, blood coagulation | *Apolipoprotein E* 🡪   - Hyperlipoproteinemia - Alzheimer disease 2 (late onset familial and sporadic form) - Sea-blue histocyte disease (splenomegaly, thrombocytopenia, histocytes in bone marrow)   *Contactin 1* 🡪 congenital myopathy (Compton-North)  *Type I collagen* 🡪   - Caffey disease - Ehlers-Danlos syndrome - Osteogenesis imperfecta 1 - 4 - Osteoporosis   *Collagen alpha-1(VI) chain* 🡪   - Bethlem myopathy 1 - Ullrich congenital muscular dystrophy   *Amyloid-beta precursor protein* 🡪   - Alzheimer disease 1 (familial early-onset form) - Cerebral amyloid angiopathy, APP-related   *Fibrinogen alpha* (and) *gamma chain* 🡪   - Congenital afibrinogenemia (tendency to bleeding and thrombosis) - Congenital dysfibrinogenemia (tendency to bleeding and thrombosis) - Amyloidosis 8 (no CNS involvement – only *fibrinogen alpha chain*)   *Alpha-2-antiplasmin* 🡪 Alpha-2-plasmin inhibitor deficiency (severe hemorrhagic diathesis) |
| Priola et al  Comparative study  (2015) | **Aim**  Proteomic alterations in CSF correlated with thromboembolic events induced by chemotherapy.  **Cohort (age range: 1 – 8 years)**  B-ALL (n=2)  T-ALL (n=2)  Treatment protocol: AALL1131 (B-ALL) and AALL0434 (T-ALL) – not further elaborated.  CSF sampled on day 0, 8 and 29. One B-ALL patient developed superior sagittal sinus, left transverse sinus and right cortical vein thromboses at day 59. | (1)  In total, 35 proteins were expressed differently between day 0, 8 and 29 (ANOVA, p<0.05) with varying intensity:  **Cluster 1** (downregulated day 0 and day 29, upregulated day 8):  *Cytoplasmic actin 2, A disintegrin and metalloproteinase with thrombospondin motifs 9, Profilin-1, Catalase, Probable phospholipid-transporting ATPase IIB* (unreviewed)*, Nestin, Carbonic anhydrase 1, Sodium/potassium-transporting ATPase subunit alpha-3, Probable ATP-dependent RNA helicase DDx41, Lithostathine-1-beta, Triosephosphate isomerase*  **Cluster 2** (upregulated day 0 and day 8, downregulated day 29):  *Ceruloplasmin, Spectrin beta chain brain 2, EGF-containing fibulin-like extracellular matrix protein 1, Probable E3 ubiquitin-protein ligase HERC2, Urokinase-type plasminogen activator, Leucine-rich alpha-2-glycoprotein, Fetuin-B, Protein unc-13 homolog B, Complement C1q subcomponent subunit C*  **Cluster 3** (downregulated day 0 and day 8, upregulated day 29):  *Mitochondrial leucine-rich PPR motif-containing protein, extracellular superoxide dismutase, RING finger and WD repeat domain-containing protein 3, Heparin cofactor 2, Apolipoprotein A-IV, POTE ankyrin domain family member F, Plasma serine protease inhibitor, Calbindin, Macrophage colony-stimulating factor 1 receptor* and*, Insulin-like growth factor-binding protein 4.*  (2)  The CSF-proteome (day 0+8+29 pooled) of the patient (n=1) with thrombosis harbored significantly different expressions of proteins compared to patients (n=3) without thromboembolic events (day 0+8+29 pooled) (t-test, p<0.05). These comprised: *Plasma serine protease inhibitor, Plasma protease C1 inhibitor, Plasminogen* and, *Factor X.* | (1)  **Day 0**  **Upregulated**  Oxidoreductase, actin-capping (probably important in neuronal membrane skeleton), growth factor, fibrinolysis, neutrophil degranulation, metalloenzyme inhibitor, and exocytosis (synaptic vesicle)  **Downregulated**  Protease, actin-binding, peroxidase, neurogenesis, lyase, translocase, helicase, neuronal sprouting, gluconeogenesis, DNA-binding, antioxidant, autophagy, hemostasis, heparin-binding, fertilization, metal-binding, inflammatory response and growth factor binding    **Day 8**  **Upregulated**  Protease, actin-binding, peroxidase, neurogenesis, lyase, translocase, helicase, neuronal sprouting, gluconeogenesis, oxidoreductase, actin-capping (probably important in neuronal membrane skeleton), growth factor, fibrinolysis, neutrophil degranulation, metalloenzyme inhibitor, exocytosis (synaptic vesicle)  **Downregulated**  DNA-binding, antioxidant, autophagy, hemostasis, heparin-binding, fertilization, metal-binding, inflammatory response, growth factor binding  **Day 29**  **Upregulated**  DNA-binding, antioxidant, autophagy, hemostasis, heparin-binding, fertilization, metal-binding, inflammatory response, and growth factor binding  **Downregulated**  Protease, actin-binding, peroxidase, neurogenesis, lyase, translocase, helicase, neuronal sprouting, gluconeogenesis, oxidoreductase, actin-capping (probably important in neuronal membrane skeleton), growth factor, fibrinolysis, neutrophil degranulation, metalloenzyme inhibitor, exocytosis (synaptic vesicle)  (2)  **Downregulated in patient with central vein thrombosis**  Heparin-binding, fertilization, blood coagulation, fibrinolysis | *Cytoplasmic actin 2* 🡪   - Deafness, autosomal dominant 20 - Baraitser-Winter syndrome 2   *Profilin-1* 🡪 Amyotrophic lateral sclerosis 18  *Catalase* 🡪 Acatalasemia  *Sodium/potassium-transporting ATPase subunit alpha-3* 🡪   - Dystonia 12 (autosomal dominant dystonia-parkinsonism disorder) - Alternating hemiplegia of childhood 2 - Cerebellar ataxia, areflexia, pes cavus, optic atrophy, and sensorineural hearing loss   *Probable ATP-dependent RNA helicase DDx41* 🡪 Myeloproliferative/lymphoproliferative neoplasms (familial – cancer predisposition syndrome, autosomal dominant inherited)  *Triosephosphate isomerase* 🡪 Triosephosphate isomerase deficiency (congenital hemolytic anemia and progressive neuromuscular dysfunction)  *Ceruloplasmin* 🡪 Aceruloplasminemia (characterized by iron accumulation in the brain)  *Spectrin beta chain brain 2* 🡪   - Spinocerebellar ataxia 5 (SCA) - Spinocerebellar ataxia, autosomal recessive, 14   *EGF-containing fibulin-like extracellular matrix protein 1* 🡪 Doyne honeycomb retinal dystrophy  *E3 ubiquitin-protein ligase HERC2* 🡪 Mental retardation, autosomal recessive 38  *Urokinase-type plasminogen activator* 🡪 Quebec platelet disorder (bleeding disorder)  *Mitochondrial leucine-rich PPR motif-containing protein* 🡪 Leigh syndrome French-Canadian type (necrotic lesions in subcortical brain)  *RING finger and WD repeat domain-containing protein 3* 🡪 Microcephaly 18, primary, autosomal dominant (brain weight markedly reduced, cerebral cortex disproportionately small)  *Heparin cofactor 2* 🡪 Thrombophilia due to heparin cofactor 2 deficiency  *Macrophage colony-stimulating factor 1 receptor* 🡪   - Leukoencephalopathy, diffuse hereditary, with spheroids (progressive neurodegenerative disorder) - Brain abnormalities, neurodegeneration, and dysosteosclerosis (progressive neurodegenerative disorder) |
| Brain tumors | | | | |
| Reichl et al  Comparative study  (2020) | **Aim**  To quantify CSF-proteome of recurrent medulloblastoma  **Cohort**  Recurrent cases of medulloblastoma prior to treatment (n=8)  Age-matched nonneoplastic controls (n=7, not further elaborated) | In comparison to the CSF-proteome of controls, there were significantly up- and downregulated proteins detected for recurrent medulloblastoma.  **Downregulated:** *Osteopontin, MAP kinase-activated protein kinase 2, Progesterone-induced-blocking factor 1, Latent-transforming growth factor beta-binding protein 4, Calmodulin-like protein 5, Protein S100-A9* and*, Protein S100-A8.*  **Upregulated:** *Kallistatin, Voltage-dependent anion-selective channel protein 1, Prolactin-inducible protein, Thioredoxin, Neuromodulin, Calbindin, Fibromodulin, Collagen alpha-3(VI) chain, Transforming growth factor-beta-induced protein ig-h3, Glypican-1, Ecto-ADP-ribosyltransferase 3, Follistatin-related protein 5, Gamma-enolase, N(G),N(G)-dimethylarginine dimethylaminohydrolase 1, Annexin A1, Dermcidin, Brain acid soluble protein 1, Cystatin-B, SH3 domain-binding glutamic acid-rich-like protein, Chitotriosidase-1, Prosaposin receptor GPR37, ADAM DEC1, A disintegrin and metalloproteinase with thrombospondin motifs 1, Annexin A5* and*, Disintegrin and metalloproteinase domain-containing protein 10* | **Downregulated**  Biomineralization, kinase (DNA damage), immunity, growth factor binding, metal binding, apoptosis, autophagy, inflammatory response  **Upregulated**  Protease inhibitor, platelet degranulation, apoptosis, actin-binding, electron transport, calmodulin-binding, developmental protein (neurogenesis), metal binding, collagen fibrillogenesis, cell adhesion, sensory transduction, glycosyltransferase, glycolysis, cell differentiation, hydrolase, inflammatory response, antibiotic, carbohydrate metabolism and Notch signaling pathway | *Progesterone-induced-blocking factor 1 🡪* Joubert syndrome 33 (cerebellar ataxia, oculomotor apraxia, hypotonia, neonatal breathing abnormalities and psychomotor delay)  *Latent-transforming growth factor beta-binding protein 4* 🡪   - Urban-Rifkin-Davis syndrome (disrupted pulmonary, gastrointestinal, urinary, musculoskeletal, craniofacial and dermal development) - Duchenne muscular dystrophy   *Collagen alpha-1(VI) chain* 🡪   - Bethlem myopathy 1 - Ullrich congenital muscular dystrophy   *Transforming growth factor-beta-induced protein ig-h3* 🡪 Corneal dystrophy (involved in seven different subtypes)  *Glypican-1* 🡪 Alzheimer disease  *Cystatin-B* 🡪 Epilepsy, progressive myoclonic 1  *Annexin A5* 🡪 Pregnancy loss, recurrent  *Disintegrin and metalloproteinase domain-containing protein 10* 🡪   - Reticulate acropigmentation of Kitamura - Alzheimer disease 18 |
| Rajagopal et al  Comparative study  (2011) | **Aim**  To investigate putative CSF-proteome biomarkers in medulloblastoma patients  **Cohort**  Medulloblastoma (n=33)  Controls (n=25): age-matched, “leftover samples drawn for other clinical purposes” | In total, 160 protein spots were on average detected in 2-DE gels, corresponding to 25 unique proteins identified by mass spectrometry. Of these, 3 proteins were significantly altered between medulloblastoma and controls (t-test or ANOVA (not specified), p<0.01).  Downregulated in medulloblastoma: *prostaglandin-H2 D-isomerase* and*, apolipoprotein E*  Upregulated in medulloblastoma:  *Clusterin* | **Downregulated**  Fatty-acid synthesis and cholesterol metabolism  **Upregulated**  Apoptosis | *Apolipoprotein E* 🡪   - Hyperlipoproteinemia - Alzheimer disease 2 (late onset familial and sporadic form) - Sea-blue histocyte disease (splenomegaly, thrombocytopenia, histocytes in bone marrow) |
| de Bont  Comparative study  (2006) | **Aim**  To detect differences in protein expression profiles of CSF from pediatric patients with and without brain tumors.  **Cohort**  Brain tumor (n=32; medulloblastoma [n=16]; high-grade glioma [n=7]; atypical rhabdoid tumor [n=2]; pilocytic astrocytoma [n=2]; plexus carcinoma [n=2]; anaplastic ependymoma [n=2]; germ cell tumor [n=1])  Control (n=70; lumbar puncture of pediatric patients 1 year after treatment of ALL [n=47]; infection [n=6]; hematological disease [n=4]; autoimmune disease [n=2]; idiopathic intracranial hypertension [n=1]; extra-CNS Hodgkin’s [n=6]; neuroblastoma [n=4]) | In total, 123 protein peak clusters were significantly different between brain tumor and controls.  One protein cluster peak was highly abundant in all brain tumor samples but virtually absent in control patients, which was identified as *apolipoprotein A-II* in mass spectrometry. | **Upregulated**  Stabilized HDL metabolism | NA |
| Spreafico et al  Comparative study  (2017) | **Aim**  To characterize the CSF proteome of patients with CSF tumors to identify biomarkers predictive of metastatic spread  **Digestion:** In solution digestion  **Cohort**  Brain tumor (n=27, medulloblastoma [n=18]; grade II and III ependymoma [n=1 and 4]; PNET [n=2]; ATRT [n=1]; high-grade glioma [n=1])  Controls: extra-CNS non-Hodgkin’s lymphoma (n=13) | In total, 51 proteins were significantly (Fisher’s exact test and/or univariate logistic regression, p<0.05 – not elaborated further) expressed between brain tumor patients and controls. The authors selected 12 proteins based on subjective assessment of relevancy. Another eight proteins were added (test, p<0.1 – as before). There was no description whether the proteins were up- or downregulated.  There was no reported difference in protein expression between the CSF-proteome of metastatic *versus* non-metastatic brain tumors.  For the Discovery cohort *versus* controls:  (1) proteins expressed (p<0.05): *Fibrinogen alpha chain (both alpha and gamma chain),* *Inter-alpha-trypsin inhibitor heavy chain H4, Collagen alpha-1(XVIII) chain,* *Insulin-like growth factor-binding protein 4, Histidine-rich glycoprotein, Procollagen C-endopeptidase enhancer 1, Collagen alpha-1(I) chain, Collagen alpha-2(I) chain,* *Selenoprotein P,* *GDNF family receptor alpha-2* and*, Immunoglobulin superfamily member 8*  (2) proteins expressed (p<0.1): *Ribonuclease pancreatic, Insulin-like growth factor-binding protein 6, Melanoma-derived growth regulatory protein, Plasma kallikrein, Complement factor D, Elastin* and, *Beta-galactoside alpha-2,6-sialyltransferase 1* | Intensity not reported. | *Fibrinogen alpha* (and) *gamma chain* 🡪   - Congenital afibrinogenemia (tendency to bleeding and thrombosis) - Congenital dysfibrinogenemia (tendency to bleeding and thrombosis) - Amyloidosis 8 (no CNS involvement – only *fibrinogen alpha chain*)   *Collagen alpha-1(XVIII) chain* 🡪 Knobloch syndrome 1 (a developmental disorder primarily characterized by typical eye abnormalities)  *Histidine-rich glycoprotein* (deficiency) 🡪 thrombophilia  *Type I collagen* 🡪   - Caffey disease - Ehlers-Danlos syndrome - Osteogenesis imperfecta 1 - 4 - Osteoporosis   *Collagen alpha-2(I) chain* 🡪   - Ehlers-Danlos syndrome, arthrochalasia type and cardiac valvular type - Osteogenesis imperfecta 1 – 4   *Plasma kallikrein* 🡪 Prekallikrein deficiency (blood coagulation defect)  *Complement factor D* 🡪 Complement factor D deficiency (susceptibility for bacterial infection)  *Elastin* 🡪   - Cutis laxa, autosomal dominant, 1 - Supravalvular aortic stenosis |
| Saratsis et al  Comparative study  (2012) | **Aim**  Exploratory study of CSF proteome in pediatric glioma patients  **Cohort**  DIPG (n=10)  GBM (n=1)  Age-matched controls (n=4, not further elaborated) | In total, 97 proteins were unique to homogenous-appearing DIPG, 124 unique to pontine gliomas with focal necrosis, and 79 shared between these 2 subtypes.  Seventy-three proteins (22%) were upregulated in 3 or more DIPG specimens, including *N(G),N(G)-dimethylarginine dimethylaminohydrolase 1*, *Peptidyl-prolyl cis-trans isomerase A*, *Tubulin beta chain*, *Vimentin*, and “members of the 14-3-3 protein family”. | **Upregulated**  Hydrolase, apoptosis, cell division, | *Tubulin beta chain* 🡪   - Cortical dysplasia, complex, with other brain malformations 6 (a disorder of aberrant neuronal migration and disturbed axonal guidance) - Skin creases, congenital symmetric circumferential, 1 (an autosomal dominant disease characterized by folded skin)   *Vimentin* 🡪 Cataract 30, multiple types |
| Bruschi et al.  Comparative study  (2021) | **Aim**: Putative biomarkers for specific brain tumor subtypes  **Digestion**: in solution  **Cohort**  Pilocytotic astrocytoma (n=8)  Gangliocytoma (n=3)  Medulloblastoma (n=7),  AT/RT (n=2)  PNET (n=1)  *Other* (n=8)  Controls, congenital hydrocephalus (n=17) | 1789 proteins were identified including 1335 proteins identified in both control and tumor samples and 263 proteins observed solely in control samples and 191 proteins observed only in tumor samples.  741 proteins were quantified in at least 70% of all analyzed samples.  241 proteins were found differentially expressed with 228 proteins enriched in control CSF and 13 proteins in tumor CSF.  *TATA-binding protein-associated factor 2N* (downregulated in tumor) and S100-B (upregulated in tumor) were the most promising proteins in CSF differentiating tumor samples from controls.  Thymosin beta-4 and CD109 could differentiate low grade gliomas and glioneural tumor samples from the other tumor samples (upregulated in both cases).  Upregulated 14-3-3 protein zeta/delta and Heat shock protein HSP 90-alpha could differentiate embryonal tumors from all other tumors. | **Upregulated in tumor**  Metal-ion binding, CNS development, actin monomer binding and serine proteases (for low grade glioma and glioneural cells *versus* other CNS tumors), and finally cadherin binding and hydrolase (for embryonal tumors *versus* other CNS tumors)  **Downregulated in tumor**  DNA binding | *TATA-binding protein-associated factor 2N* 🡪 Translocation t(9;17)(q22;q11) with NR4A3 is linked to extraskeletal myxoid chondrosarcomas |

Abbreviations: 2-DE (two-dimensional gel electrophoresis), ALL (acute lymphoblastic leukemia), CNS (central nervous system), CSF (cerebrospinal fluid), ITC (intrathecal chemotherapy), MRD (Minimal Residual Disease), PCR (polymerase chain reaction), DIPG (diffuse intrinsic pontine glioma). We omitted *Findings* of “uncharacterized proteins” and hemoglobin (considered blood contaminants).
